# Genome-Wide Characterisation of Somatic Mutations Across COSMIC Cancer Cell Lines Enables Gene-Centric Experimental Model Selection

**DOI:** 10.3390/ijms27198636

**Published:** 2026-09-27

**Authors:** Ali F. Alsulami

**Affiliations:** Department of Biochemistry, Faculty of Science, King Abdulaziz University, Jeddah 21589, Saudi Arabia; afmalsulami1@kau.edu.sa

**Keywords:** COSMIC Cell Lines Project, cancer cell lines, somatic mutations, Cancer Gene Census, hotspot mutations, experimental model selection, precision oncology

## Abstract

Cancer cell lines are widely used to investigate cancer-associated mutations and evaluate candidate therapeutic compounds, but selecting an appropriate model carrying a specific mutation can be challenging. A genome-wide analysis of somatic mutations across 1020 human cancer cell lines from the COSMIC Cell Lines Project was performed to establish a gene- and variant-centric approach for experimental model selection. Transcript-specific annotations were normalised to gene symbols, and redundant transcript-derived records were removed. The resulting dataset contained 3,937,879 unique somatic mutation events affecting 18,885 genes, including 95,307 mutation events across 579 Cancer Gene Census Tier 1 genes. Substantial variation in mutation burden was observed among cell lines, while recurrent cancer-associated genes, including *TP53*, *KRAS*, *BRAF*, *EGFR* and *PIK3CA*, were represented across multiple models. Importantly, the cell line containing the greatest number of alterations in a cancer-associated gene did not necessarily carry the specific driver mutation required for a particular experimental question. Representative driver variants were therefore used to demonstrate a variant-centric selection strategy in which cell lines carrying the specific alteration of interest are identified rather than models being selected solely according to gene-specific mutation count. This approach distinguishes descriptive gene-level mutation ranking from variant-specific experimental model selection and provides a systematic framework for identifying genetically appropriate candidate cell lines for functional studies and compound testing.

## 1. Introduction

Cancer is a complex genetic disease characterised by the progressive accumulation of somatic genomic alterations that disrupt normal cellular processes and promote malignant transformation [1]. These alterations include single-nucleotide variants [2], insertions and deletions [3], copy number abnormalities and structural rearrangements that collectively influence tumour initiation [4], progression, metastasis and therapeutic response [5,6]. Although the majority of somatic mutations represent biologically neutral passenger events, a relatively small proportion function as driver mutations that confer selective growth advantages through the disruption of critical cellular pathways controlling proliferation [7], apoptosis [8], genome stability, DNA repair and signal transduction [9]. Identifying these driver alterations and understanding their functional consequences remain central objectives of cancer biology and precision oncology [10].

The rapid development of next-generation sequencing technologies has revolutionised cancer genomics by enabling the comprehensive characterisation of tumour genomes across virtually all major cancer types [11,12]. Large international initiatives, including The Cancer Genome Atlas (TCGA) [13], the International Cancer Genome Consortium (ICGC) [14] and the Catalogue of Somatic Mutations in Cancer (COSMIC) [15], have generated extensive catalogues of somatic mutations and have substantially improved our understanding of the genomic diversity underlying human malignancies [16,17]. These resources have revealed remarkable inter- and intra-tumour heterogeneity, demonstrating that cancers arising from the same tissue frequently exhibit distinct mutational profiles and molecular mechanisms [18,19]. Consequently, accurate genomic characterisation has become fundamental for biomarker discovery, therapeutic target identification and the implementation of precision medicine.

Human cancer cell lines remain one of the most widely used experimental models for investigating tumour biology [20]. Owing to their relative ease of culture, reproducibility and genetic stability during laboratory propagation, cancer cell lines have become indispensable tools for studying oncogenic signalling pathways, tumour suppressor function, drug resistance, synthetic lethality and mechanisms of therapeutic response [21,22,23]. They are extensively used in functional genomics, CRISPR-based genetic screening [24], high-throughput drug discovery, structural biology and preclinical evaluation of targeted therapies [25]. Importantly, many widely used cancer cell lines preserve key molecular alterations found in primary tumours, making them valuable surrogate models for experimental cancer research [26,27].

Several large-scale resources have systematically characterised cancer cell lines at the genomic, transcriptomic and pharmacological levels. The COSMIC Cell Lines Project [28], the Cancer Cell Line Encyclopedia (CCLE) [29] and the Genomics of Drug Sensitivity in Cancer (GDSC) [30] collectively provide mutation profiles, gene expression data, copy number alterations and drug-response measurements for hundreds of cancer cell lines representing diverse tumour types. These publicly available resources have enabled numerous investigations into cancer evolution, therapeutic vulnerabilities and predictive biomarkers, while also facilitating the development of computational methods for drug sensitivity prediction and precision oncology. Previous studies have successfully demonstrated that many cancer cell lines closely resemble their corresponding primary tumours with respect to major driver mutations and molecular pathways, supporting their continued importance as experimental systems [31,32,33,34].

Despite these valuable resources, selecting the most appropriate cancer cell line for a particular biological question remains challenging [35]. Experimental models are frequently chosen according to tissue of origin, historical use, or laboratory availability rather than their detailed genomic characteristics. Although comprehensive mutation catalogues are publicly available, extracting biologically meaningful information from thousands of transcript-specific mutation records distributed across individual cell-line datasets is time-consuming and computationally demanding. Furthermore, mutation annotations are frequently reported at the transcript level, resulting in multiple transcript-specific entries describing the same biological mutation and potentially inflating gene-level mutation counts. These issues complicate direct comparisons among genes, pathways and cancer cell lines and may obscure biologically relevant patterns within large genomic datasets.

An additional challenge is that mutation prevalence or burden alone does not necessarily identify the most informative experimental model. Large genes such as *TTN*, *MUC16*, *HYDIN*, *NEB* and *OBSCN* accumulate large numbers of passenger mutations because of their genomic size, whereas canonical cancer driver genes may contain relatively few but highly functional hotspot mutations [36]. Likewise, the cancer cell line harbouring the greatest number of mutations within a particular gene is not always the most biologically representative model if those alterations are predominantly synonymous or non-functional [37]. Conversely, a cell line containing a single well-characterised activating mutation, such as Serine/threonine-protein kinase B-raf (*BRAF*) p.V600E, GTPase Kras (*KRAS*) p.G12D, Epidermal growth factor receptor (*EGFR*) p.L858R or Phosphatidylinositol 4,5-bisphosphate 3-kinase catalytic subunit alpha isoform (*PIK3CA*) p.H1047R, may provide a substantially more appropriate system for mechanistic studies and therapeutic evaluation [38,39,40,41]. Consequently, a comprehensive interpretation of cancer cell line mutations requires consideration of mutation burden, recurrence, biological function and the presence of clinically relevant hotspot variants rather than mutation count alone.

Previous pan-cancer analyses have primarily focused on cataloguing genomic alterations, evaluating concordance between cancer cell lines and primary tumours, or associating genomic features with drug sensitivity [29,42,43]. While these studies have substantially advanced the field, comparatively fewer investigations have integrated transcript-level normalisation, genome-wide mutation characterisation, Cancer Gene Census prioritisation and hotspot mutation-guided experimental model selection into a single analytical framework [18,44,45]. Such an approach has the potential to generate a more biologically meaningful overview of cancer cell line genomics while simultaneously providing practical guidance for selecting appropriate experimental models according to specific genes, signalling pathways and therapeutic targets.

Despite the extensive genomic resources available for cancer cell lines, an important gap remains in translating these mutation catalogues into a practical framework for experimental model selection. Existing databases primarily provide mutation annotations for individual cell lines or focus on pharmacogenomic associations, requiring researchers to manually interrogate thousands of mutation records to identify suitable models for specific genes or signalling pathways [29]. Furthermore, previous pan-cancer studies have largely emphasised mutation cataloguing rather than systematically distinguishing recurrent passenger alterations from biologically relevant driver mutations or identifying representative cell lines carrying clinically important hotspot variants [36,37,46]. As a result, selecting the most appropriate experimental model for functional studies, target validation, or drug discovery remains challenging and often depends on prior knowledge rather than systematic genomic evidence.

To address this gap, this study presents a comprehensive genome-wide comparative analysis of somatic mutations across the COSMIC Cell Lines Project with the aim of establishing a practical, gene-centric approach for cancer cell-line selection. Integrating mutation burden, gene recurrence, Cancer Gene Census annotations and canonical hotspot mutations within major cancer hallmark pathways, the study provides a systematic framework for prioritising genetically relevant experimental models. Rather than simply cataloguing mutations, this work links genomic alterations to representative cell lines, enabling researchers to rapidly identify appropriate in vitro models for mechanistic investigations, biomarker discovery, therapeutic target validation and precision oncology research.

## 2. Results

A comprehensive comparative analysis of the COSMIC Cell Lines Project was performed following transcript-level normalisation, whereby transcript-specific isoforms were collapsed into their corresponding gene symbols, and redundant transcript-derived mutation records were removed. The final curated dataset comprised 1020 human cancer cell lines, representing 3,937,879 unique somatic mutation events distributed across 18,885 unique genes, including 95,307 mutations affecting 579 Cancer Gene Census (CGC) Tier 1 genes (Supplementary Materials).

Substantial heterogeneity in mutation burden was observed among individual cancer cell lines (Figure 1A). The hypermutated cell line SNU-1040 exhibited the largest number of unique mutation events (98,200), followed by Daudi (67,635), HCC2998 (56,793), SNU-81 (56,687) and CW-2 (45,113). In contrast, numerous commonly used cancer cell models contained considerably fewer mutations, demonstrating the extensive genomic diversity represented within the COSMIC cell line collection. Similarly, the number of unique mutated genes varied markedly among cell lines (Figure 1B), indicating that cancer models differ not only in mutation burden but also in the breadth of genes affected by somatic alterations.

Analysis of mutation classes demonstrated that single nucleotide substitutions dominated the mutational landscape (Figure 2A). Missense mutations represented the largest functional mutation class, accounting for 863,041 unique mutation events, followed by 433,165 synonymous substitutions. Protein-truncating alterations occurred less frequently, including 49,196 nonsense mutations and 46,555 frameshift deletions, while frameshift insertions, in-frame insertions, in-frame deletions and other complex mutation classes collectively represented a smaller proportion of the dataset. The predominance of missense mutations indicates that amino acid substitutions constitute the principal source of protein sequence variation across cultured cancer cell models.

Because mutations classified as “Unknown” represented a substantial proportion of the dataset, a sensitivity analysis was performed to determine whether their inclusion influenced cell-line mutation-burden rankings. Of the 3,937,879 unique somatic mutation events, 2,513,401 (63.83%) were classified as Unknown. Exclusion of these events left 1,424,478 mutation events with an annotated mutation type. The cell-line ranking obtained after excluding Unknown events showed a very strong correlation with the primary ranking based on all mutation events (Spearman’s ρ = 0.983, *p* < 0.001). Eighteen of the 20 highest-ranked cell lines in the primary analysis remained among the top 20 after exclusion of Unknown events. SNU-1040 and Daudi remained the two highest-ranked cell lines, with 43,476 and 40,881 annotated mutation events, respectively. Although some changes in rank were observed, the overall ranking pattern remained highly consistent, indicating that the principal cell-line mutation-burden rankings were robust to exclusion of mutations classified as Unknown (Table 1).

Genome-wide analysis identified 18,885 mutated genes; however, mutation recurrence varied substantially between genes (Figure 2B). Several exceptionally large genes, including *TTN*, *HYDIN*, *MUC16*, *MUC4*, *NEB*, *OBSCN* and *SYNE1*, accumulated the greatest numbers of mutation events. The high numbers of mutation events observed in these genes are likely influenced by their large genomic size, which increases the opportunity for passenger alterations. When recurrence was assessed according to the number of independent cell lines harbouring mutations (Figure 3, *TTN* remained the most frequently altered gene, being mutated in 930 of 1020 cell lines (91.2%). Importantly, well-established cancer driver genes were also highly recurrent, with TP53 mutated in 718 cell lines (70.5%), followed by numerous chromatin-remodelling and tumour suppressor genes that were altered across hundreds of independent cancer models.

Examination of chromosomal mutation distribution demonstrated that somatic mutations were detected on every autosome and the X chromosome, although the number of observed mutation events varied considerably among chromosomes (Figure 4A). Chromosome 1 harboured the largest number of mutations (388,747), followed by chromosomes 2 (329,741) and 3 (238,503). High mutation burdens were also observed on chromosomes 19, 11, 12, 5 and 7, whereas the fewest mutations occurred on chromosomes 21, 18, 13 and 22. The X chromosome contained 105,221 mutation events, while only 1305 mutations were identified on the Y chromosome. Overall, mutation burden broadly reflected chromosome size and gene density, with larger, gene-rich chromosomes generally accumulating more mutations. Notably, chromosome 19 displayed a disproportionately high number of observed mutation events relative to its size, consistent with its exceptionally high gene density.

Analysis of amino acid substitution patterns further demonstrated that a limited number of residue changes accounted for a substantial proportion of protein-coding variation across the dataset (Figure 3). Analysis of amino-acid substitution patterns further demonstrated that a limited number of residue changes accounted for a substantial proportion of protein-coding variation across the dataset (Figure 3). Recurrent amino-acid substitutions were observed across multiple cancer cell lines, demonstrating that certain residue changes recur within the analysed dataset. Together, these findings demonstrate that although cancer cell lines exhibit substantial genomic heterogeneity, they share recurrently mutated genes, recurrent amino-acid substitution patterns and characteristic chromosomal mutation distributions that collectively describe the mutational landscape of the analysed cancer cell models.

To focus on clinically relevant cancer-associated alterations, recurrent mutations were further examined within Cancer Gene Census (CGC) Tier 1 genes (Figure 3). A total of 95,307 unique mutation events were identified across 579 Tier 1 genes, demonstrating that virtually all major oncogenic signalling pathways are represented within the COSMIC cell line collection. *TP53* was the most recurrent Tier 1 gene, with 880 unique mutation events distributed among 718 independent cell lines (70.4%), substantially exceeding all other canonical cancer genes. Interestingly, despite its widespread occurrence, no individual cell line contained more than four unique TP53 mutation events, indicating that TP53 alterations are broadly distributed throughout the COSMIC collection rather than being concentrated within a small number of hypermutated models. The highest-ranked *TP53* models included GCT, KARPAS-45, MCC13, PCI-4B, SKG-IIIa, SKN-3 and SNU-1040, each harbouring four independent TP53 mutations comprising missense, nonsense and synonymous substitutions, thereby providing genetically diverse experimental systems for investigating p53 dysfunction and DNA damage response pathways.

In contrast, several other Tier 1 genes exhibited pronounced mutation enrichment within individual cell lines. LRP1B, the second most recurrent Tier 1 gene, was mutated in 610 cell lines, with SNU-81 and HCC2998 each containing 42 unique mutation events, followed by Mewo (37 mutations) and SNU-1040 (30 mutations). Similarly, PDE4DIP mutations were identified in 589 cell lines, although SNU-1040 alone accumulated 30 distinct mutation events. Comparable enrichment patterns were observed for KMT2C (526 mutated cell lines; 26 mutations in SNU-1040) and TRRAP (433 mutated cell lines; 32 mutations in SNU-1040), highlighting that while *TP53* mutations are widely dispersed across cancer models, alterations in several chromatin-remodelling and genome-maintenance genes tend to accumulate within specific hypermutated cell lines. These findings demonstrate that recurrent Tier 1 mutations differ not only in their prevalence across cancer cell lines but also in their distribution among individual models, emphasising the importance of considering both mutation recurrence and mutation burden when selecting appropriate experimental systems for functional studies (Table 2).

To further enhance the practical utility of the COSMIC cell-line resource, representative experimental models were identified for major cancer hallmark genes by integrating mutation burden with the presence of well-established oncogenic hotspot mutations (Table 2). Although the initial ranking was based on the number of unique mutation events observed for each gene in individual cell lines, closer inspection revealed that the most mutation-rich model was not necessarily the most biologically informative (Supplementary Materials). For example, Farage contained the highest number of *BRAF* mutation records; however, these variants did not include the canonical activating p.V600E substitution. In contrast, multiple melanoma and colorectal cancer cell lines, including A375, LOXIMVI, HT-29, ACN, BHT-101, IGR-1 and LS-411N, harboured the clinically relevant *BRAF* p.V600E mutation and therefore represent more appropriate experimental models for investigating BRAF-driven oncogenesis and evaluating *BRAF*-targeted therapies. A similar pattern was observed for KRAS, where the highest-ranked model (Daudi) accumulated several *KRAS* variants but lacked the classical activating hotspot mutations. Instead, functionally important alterations such as p.G12D, p.G12V, p.G12C and p.G13D were identified in established experimental models including GP5d, AsPC-1, CFPAC-1, CAPAN-1 and NCI-H2291, which are more suitable for studies of mutant KRAS signalling and targeted inhibition. Likewise, clinically actionable EGFR mutations, including exon 19 deletions and p.L858R, were identified in HCC827, PC-14 and other lung cancer models, while activating *PIK3CA* p.E545K and p.H1047R mutations were detected in representative breast, colorectal and oesophageal cancer cell lines. These observations demonstrate that quantitative mutation burden alone is insufficient for selecting experimental models and highlight the importance of considering the biological significance of individual variants. Consequently, Table 3 combines gene recurrence, mutation burden and canonical hotspot mutations to provide a practical guide for selecting genetically relevant cell lines for mechanistic studies, drug discovery and precision oncology research.

## 3. Discussion

The present study provides a genome-wide characterisation of somatic mutations across the COSMIC Cell Lines Project and demonstrates how large-scale mutation data can support experimental model selection [15]. Transcript-level normalisation and deduplication established a non-redundant gene-level representation of the data, while cross-cell-line analysis, Cancer Gene Census (CGC) Tier 1 annotation, and the evaluation of canonical driver variants enabled mutations to be considered according to both their distribution and biological relevance. This approach is important because individual genes may be represented by multiple transcript-specific records and cell lines differ markedly in their overall mutation burden. Consequently, direct comparison of unprocessed mutation records may obscure biologically relevant differences among experimental models.

One of the most prominent observations was the substantial heterogeneity in somatic mutation burden across cancer cell lines. Whereas some models contained tens of thousands of alterations, others carried substantially fewer mutations, and recurrent cancer-associated alterations were distributed across a broad range of cell lines. This heterogeneity is consistent with the genomic diversity of cancer and indicates that individual cell lines represent distinct mutational backgrounds rather than interchangeable experimental models.

The marked differences in somatic mutation burden observed among individual cell lines may reflect several underlying biological processes. Hypermutated cancer genomes can arise through alterations affecting DNA replication fidelity or DNA-repair pathways; however, the mutation records analysed in the present study do not provide sufficient information to determine the mechanisms responsible for the elevated mutation burden of individual cell lines. Although sequence alterations in genes involved in genome maintenance may be present, their occurrence alone does not establish functional deficiency or demonstrate a mutator phenotype. Establishing such mechanisms would require complementary information, including mismatch-repair status, microsatellite instability, mutational signatures and functional characterisation of the relevant variants. Consequently, the high mutation burdens observed in individual cell lines should be interpreted as genomic characteristics of these models rather than evidence of a specific underlying mutator mechanism.

The variation in the number of somatic alterations among chromosomes may likewise reflect differences in their underlying genomic characteristics. Larger chromosomes provide a greater genomic sequence space in which alterations can occur and may therefore accumulate higher absolute numbers of mutation events, which may partly explain the relatively high counts observed for chromosomes 1, 2 and 3. Nevertheless, chromosome length alone is unlikely to account for the complete distribution, as chromosomes also differ in gene density, sequence composition and other genomic features that may influence the number of observed alterations. For example, chromosome 19 is relatively small but highly gene-rich, which may contribute to its comparatively high number of observed mutation events. Because the present study reports absolute mutation counts rather than chromosome-length-normalised mutation rates, these observations should be interpreted descriptively and should not be considered evidence of differences in intrinsic chromosome-specific mutability.

Gene-level analysis further demonstrated that mutation abundance should be distinguished from biological relevance. Large structural genes, including *TTN*, *MUC16*, *HYDIN* and *OBSCN*, were among the most frequently altered genes in the dataset. Their high mutation counts may be influenced substantially by their large coding sequence lengths, which provide greater opportunity for accumulation of passenger alterations. Consequently, ranking genes according to mutation counts alone can cause large genes to dominate the analysis while potentially obscuring cancer-associated drivers. The incorporation of CGC Tier 1 annotations and established driver variants therefore provides an important additional level of biological interpretation.

This distinction was particularly evident when individual cancer-associated genes were examined across cell lines. The model containing the greatest number of alterations in a gene did not necessarily carry the canonical driver variant relevant to a specific experimental question. For example, Farage contained the greatest number of BRAF alterations in the analysed dataset but did not harbour the canonical p.V600E variant, whereas A375, HT-29 and LOXIMVI carried *BRAF* p.V600E. Similar patterns were observed for *KRAS*, *EGFR* and *PIK3CA*, where established driver alterations occurred in models other than those ranked highest by total gene-specific mutation count. These observations demonstrate the value of variant-level interrogation when selecting models for studies focused on specific oncogenic alterations.

Previous efforts have substantially advanced the molecular characterisation of cancer cell lines. The Cancer Cell Line Encyclopedia and Genomics of Drug Sensitivity in Cancer have established comprehensive genomic and pharmacological datasets, while comparisons between cancer cell lines and primary tumours have demonstrated substantial variation in the extent to which individual models reproduce tumour molecular characteristics. The present study complements these resources by organising large-scale COSMIC mutation data into a gene- and variant-centric framework that facilitates the identification of genetically appropriate candidate models for individual genes and signalling pathways, thereby providing a practical link between genomic annotation and experimental design.

Several limitations should be considered when interpreting these findings. First, the analysis is based exclusively on somatic sequence alterations and does not incorporate copy-number alterations, gene expression, epigenetic regulation or protein abundance, all of which may influence cellular phenotype and pathway activity. Second, mutation recurrence or abundance does not necessarily indicate functional importance, particularly for large genes susceptible to accumulation of passenger alterations. Third, cancer cell lines undergo prolonged in vitro propagation and may acquire genomic changes that distinguish them from their tumours of origin. Finally, mutation data alone cannot establish the functional consequences of individual variants or the activity of the corresponding signalling pathways. Therefore, genomic mutation status should be considered together with tissue origin and complementary molecular and functional information when selecting an experimental model.

From an experimental perspective, the results provide guidance for selecting cancer cell lines according to the biological question being investigated. Cell lines with exceptionally high somatic mutation burdens, including SNU-1040, Daudi, HCC2998 and SNU-81, may serve as candidate models for studies specifically requiring mutation-rich genomic backgrounds, although their suitability will also depend on tissue origin and other molecular characteristics. For studies targeting particular cancer-associated pathways or mutant proteins, selection should instead focus on the specific driver alteration. For example, A375, HT-29 and LOXIMVI represent candidate models for *BRAF* p.V600E-associated studies; AsPC-1 and other KRAS p.G12D-positive models may be considered for mutant *KRAS* studies; HCC-827 and PC-14 carry EGFR p.E746_A750del; and BT-20 and CAL-148 carry *PIK3CA* p.H1047R. Thus, the appropriate experimental model depends on the molecular question being addressed, and neither overall mutation burden nor gene-specific mutation count should be used as the sole criterion for cell-line selection.

Future studies could extend this framework by incorporating additional molecular layers. Integration of mutation profiles with transcriptomic, proteomic and pharmacogenomic data could enable cell-line prioritisation based on both mutation status and pathway activity. Clinically curated knowledge bases such as OncoKB [47], CIViC [48] and the COSMIC Cancer Mutation Census 19 could further support the interpretation of cancer-associated variants. Integration with drug-sensitivity and CRISPR-dependency datasets could ultimately improve the selection of experimental models for the functional investigation and evaluation of compounds directed against specific molecular alterations.

## 4. Materials/Methods

### 4.1. Data Preprocessing and Transcript Normalisation

Somatic mutation data for 1020 human cancer cell lines were obtained from the COSMIC Cell Lines Project and processed using custom Python3.10 scripts. Column names were first standardised across individual cell-line files, and the cell-line identifier was derived from each source filename. Transcript-specific gene labels were normalised by removing the appended ENST transcript identifier and collapsing them to the corresponding gene symbol (e.g., TTN_ENST00000460472 to TTN). Records lacking a valid gene symbol after normalisation were excluded from subsequent analysis.

To remove redundant transcript-derived representations while preserving distinct annotated consequences, a composite mutation key was generated for every record using six fields: cell line, normalised gene symbol, genomic position, CDS alteration, amino-acid alteration and mutation type. Before the construction of the key, blank or missing text values were standardised as “Unknown”. Records with identical values across all six components of the composite key were considered redundant representations of the same annotated mutation event, and only the first occurrence was retained. In contrast, records occurring at the same genomic position but differing in CDS alteration, amino-acid consequence or mutation type generated different composite keys and were therefore retained as distinct annotated events. No preferred transcript was selected, and conflicting or alternative protein consequences were not computationally reconciled. This conservative procedure was designed to remove only records that became identical according to the defined composite criteria following gene-symbol normalisation.

### 4.2. Terminology and Mutation Characterization

Terminology used throughout this study refers to cross-sectional comparisons of pre-existing somatic alterations across genetically distinct cancer cell lines rather than mutation rates measured across generations. A unique mutation event was defined as a non-redundant somatic sequence alteration within an individual cell line following transcript normalisation and deduplication. Mutation burden refers to the number of unique mutation events within a specified analytical unit, whereas gene recurrence refers to the number or proportion of independent cell lines harbouring at least one alteration in a given gene. Mutation-class distribution describes the distribution of alterations among annotation classes, including missense, synonymous, nonsense, frameshift and in-frame variants. Canonical hotspot mutations refer to established recurrent cancer-associated variants at specific residues or genomic positions, such as BRAF p.V600E, KRAS codon 12 substitutions and PIK3CA p.H1047R, rather than experimentally measured sites of increased mutability.

Following preprocessing, mutation burden and the number of unique mutated genes were calculated for each cell line. Gene-level recurrence was determined from the number of independent cell lines harbouring alterations in each gene, and the total number of unique mutation events per gene was also calculated. Mutation classes and recurrent amino-acid substitutions were summarised across the dataset. Chromosomal distribution was determined from genomic coordinates by counting unique mutation events assigned to each chromosome.

Missing or blank values in the COSMIC mutation-type field were standardised as “Unknown”. Accordingly, the “Unknown” category in the mutation-class analysis comprised records explicitly annotated as “Unknown” in the source COSMIC data together with records for which the mutation-type field was blank or missing and was subsequently standardised as “Unknown” during preprocessing. This classification does not indicate that the underlying genomic alteration itself was unidentified. These records were retained in the primary dataset because they represented reported somatic alterations and could contain other sequence-level information, including genomic position, CDS alteration or amino-acid consequence. Consequently, records classified as “Unknown” were included in calculations of overall mutation burden, gene-level mutation counts and cell-line rankings, while their mutation class was represented as “Unknown” in mutation-class summaries.

To assess whether the inclusion of these records influenced cell-line mutation-burden rankings, a sensitivity analysis was performed by excluding all mutation events classified as “Unknown”. Mutation burden was recalculated independently for each of the 1020 cancer cell lines, and the resulting rankings were compared with the primary rankings based on all mutation events. Ranking agreement was evaluated using Spearman’s rank correlation coefficient (ρ), and stability among the highest-ranked models was further assessed by determining the overlap between the top 20 cell lines in the primary and sensitivity analyses.

### 4.3. Cancer-Gene and Variant-Level Analysis

Cancer Gene Census (CGC) Tier 1 annotations provided by COSMIC were used to identify genes with established roles in cancer. For each Tier 1 gene, the number of mutated cell lines, unique mutation events, amino-acid alterations, mutation classes, zygosity and validation status were summarised. Major cancer-associated genes, including *TP53*, *KRAS*, *BRAF*, *EGFR*, *PIK3CA*, *APC*, *PTEN*, *RB1*, *CDKN2A*, *ATM*, *BRCA1*, *BRCA2*, *ARID1A* and *SMAD4*, were subsequently examined at the variant level.

To demonstrate the practical application of the gene- and variant-centric model-selection framework, selected well-established cancer-associated driver variants were examined as illustrative examples. These variants were not intended to constitute a comprehensive or systematically ranked catalogue of cancer mutation hotspots. Rather, they were used to demonstrate how selection based on the presence of a specific mutation may differ from selection based solely on the absolute number of alterations in the corresponding gene. Representative variants included *KRAS* p.G12D and p.G12V, *BRAF* p.V600E, EGFR p.L858R and *PIK3CA* p.H1047R. For each selected variant, the processed dataset was queried to identify cell lines carrying the exact amino-acid alteration, and these models were compared with cell lines exhibiting high gene-specific mutation counts. The resulting examples were used to illustrate the distinction between gene-level descriptive ranking and variant-specific experimental model selection (Table 2).

### 4.4. Gene- and Variant-Centric Cell-Line Prioritisation

To provide a reproducible strategy for experimental model selection, candidate cell lines were evaluated using a hierarchical decision framework rather than an additive numerical scoring system (Figure 1). The framework was designed to identify cell lines carrying the molecular alteration required by the experimental question rather than to rank models solely according to gene-specific or genome-wide mutation burden.

When a specific cancer-associated variant was predefined, cell lines carrying the exact alteration were identified and prioritised over models containing other alterations in the same gene, irrespective of their absolute gene-specific or overall mutation burden. Genome-wide mutation burden was considered an important contextual characteristic rather than an exclusion criterion. Consequently, a hypermutated cell line was not automatically excluded when it carried the required driver variant, particularly when that alteration was not represented in another available model. When multiple cell lines carried the same target variant, the overall mutation burden could be considered as an additional characteristic for distinguishing among otherwise genetically suitable candidate models.

When no specific variant was predefined, the analysis proceeded at the gene level. Cell lines harbouring alterations in the target gene were identified, with CGC Tier 1 annotation used to establish recognised cancer-gene relevance and recurrence across independent cell lines used to describe the representation of the altered gene across available models. Gene-specific and overall mutation burdens were retained as descriptive and contextual measures and were not interpreted independently as indicators of experimental suitability.

Thus, the framework distinguishes gene-level descriptive ranking from variant-specific experimental model selection. A high number of alterations in a cancer-associated gene does not by itself establish that a cell line is the most appropriate experimental model. Conversely, a highly mutated cell line may remain a relevant candidate when it carries the specific driver alteration required by the experimental question. The final model selection should additionally consider tissue of origin, cancer type, and other molecular or phenotypic characteristics where such information is available, as these factors were not systematically represented in the mutation dataset analysed here. The complete prioritisation procedure is summarised in Figure 5.

## 5. Conclusions

In this study, a comprehensive genome-wide characterisation of somatic mutations was performed across 1020 human cancer cell lines from the COSMIC Cell Lines Project, and a standardised computational framework was developed for large-scale comparative mutation analysis. By collapsing transcript-specific gene annotations into unified gene symbols and removing redundant mutation records, we generated a curated dataset that enabled systematic evaluation of mutation burden, recurrently mutated genes, mutation classes, chromosomal mutation distribution, recurrent amino acid substitutions and Cancer Gene Census Tier 1 genes across a diverse collection of cancer models.

Beyond providing a global overview of the mutational landscape, this study introduces a practical gene-centric strategy for selecting representative experimental cell lines. Importantly, we demonstrate that mutation abundance alone is insufficient for model selection, as the cell line containing the largest number of mutations within a gene does not necessarily harbour the biologically relevant or clinically actionable hotspot mutation. By integrating mutation recurrence with canonical driver mutations across major cancer hallmark genes, including *TP53*, *KRAS*, *BRAF*, *EGFR*, *PIK3CA*, *APC*, *PTEN*, *RB1*, *CDKN2A*, *ATM*, *BRCA1*, *BRCA2* and *ARID1A*, the proposed framework provides a biologically informed resource for identifying appropriate in vitro models for mechanistic studies, target validation and therapeutic development.

## Figures and Tables

**Figure 1:**
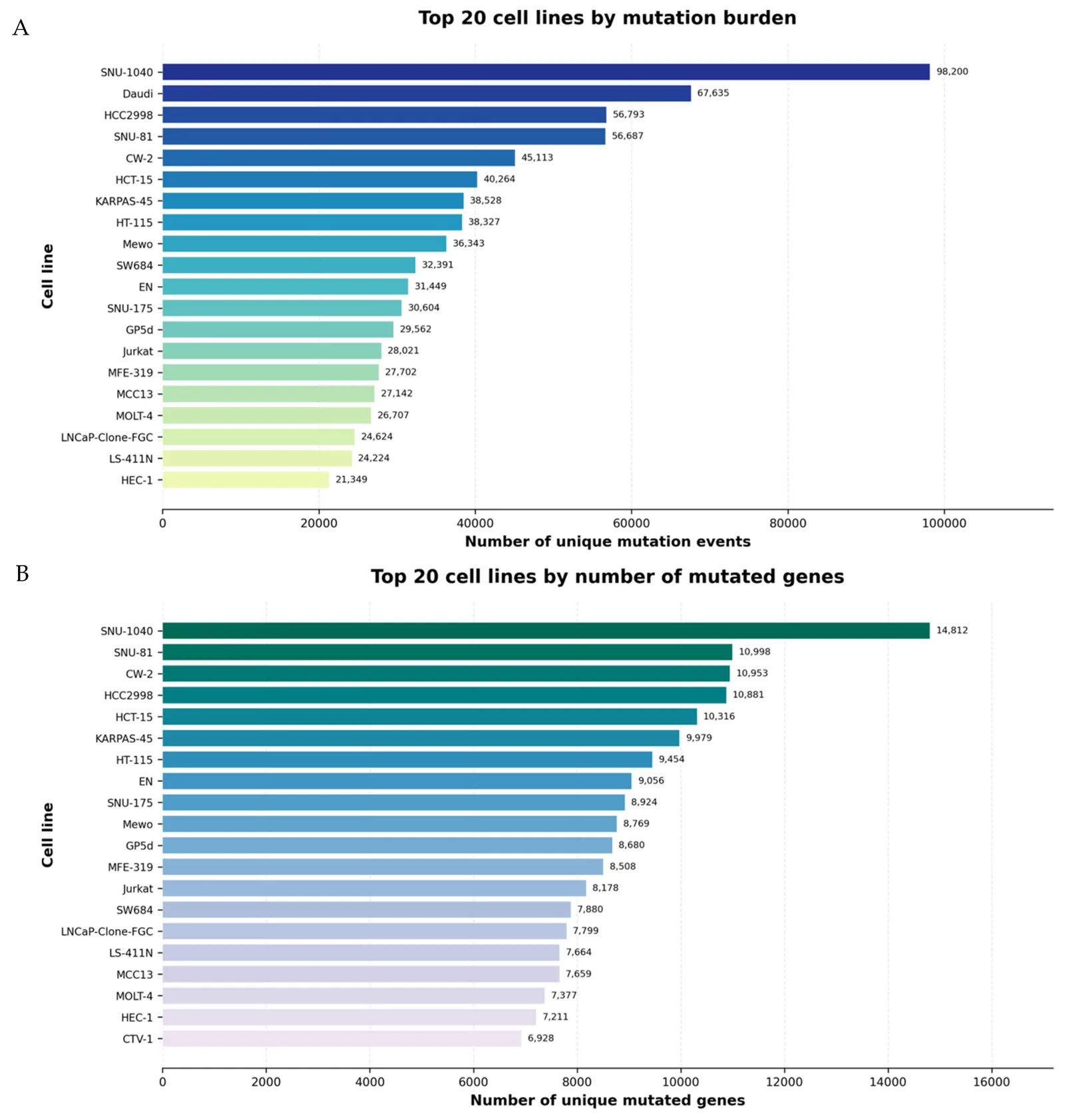
Genome-wide mutation burden across the COSMIC Cell Lines Project. (**A**) Top 20 cancer cell lines ranked according to the total number of unique somatic mutation events. (**B**) Top 20 cancer cell lines ranked according to the total number of unique mutated genes. The values shown on each bar represent the corresponding mutation counts.

**Figure 2:**
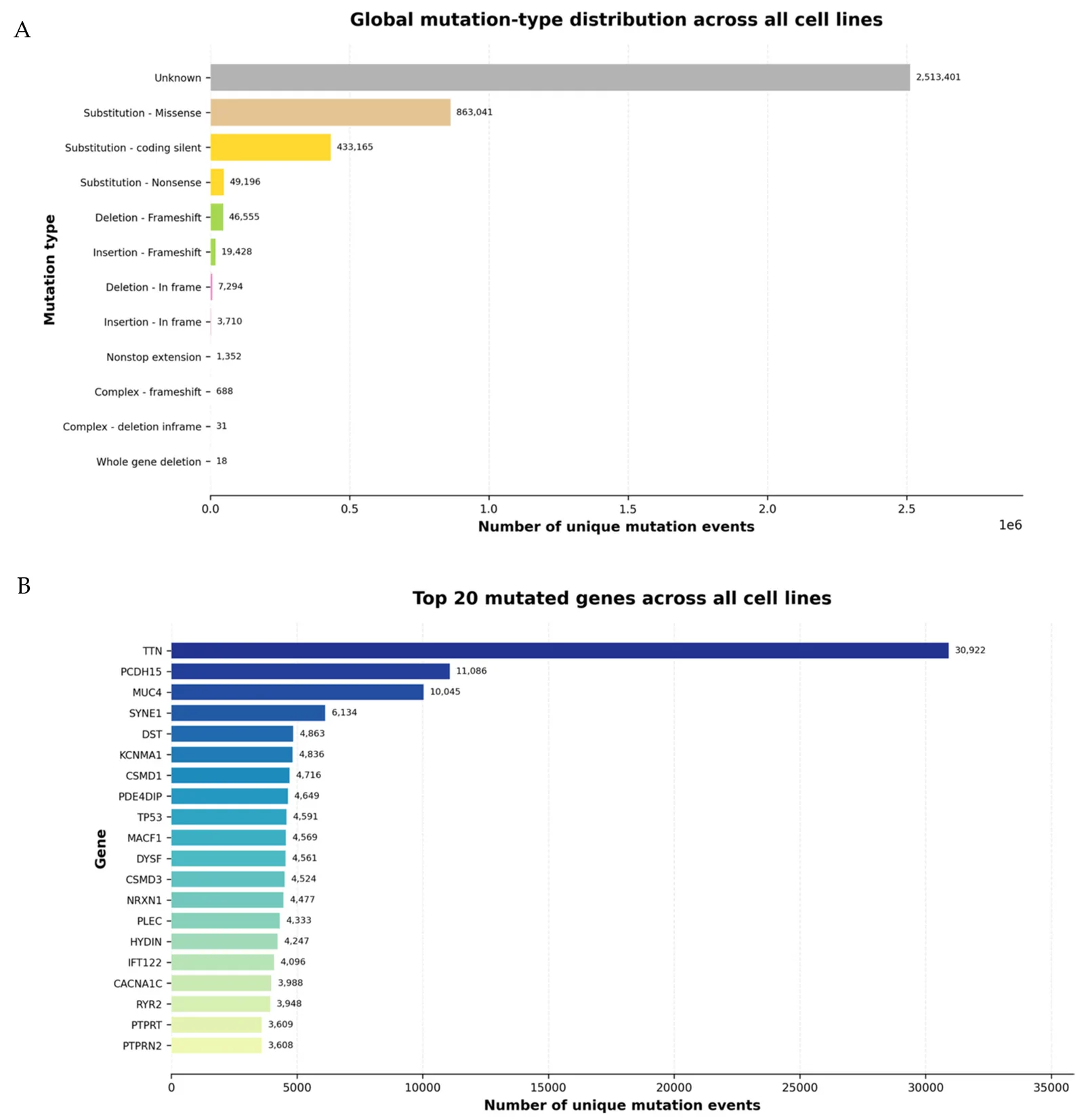
Distribution of mutation classes and recurrently mutated genes across the COSMIC Cell Lines Project. (**A**) Distribution of mutation classes identified across all analysed cancer cell lines. Unknown denotes mutation records lacking a specified mutation-type annotation in the source COSMIC data; these records were retained in mutation-burden calculations. (**B**) Top 20 genes ranked according to the total number of unique mutation events observed across the complete dataset. Values shown on the bars indicate the corresponding mutation counts.

**Figure 3:**
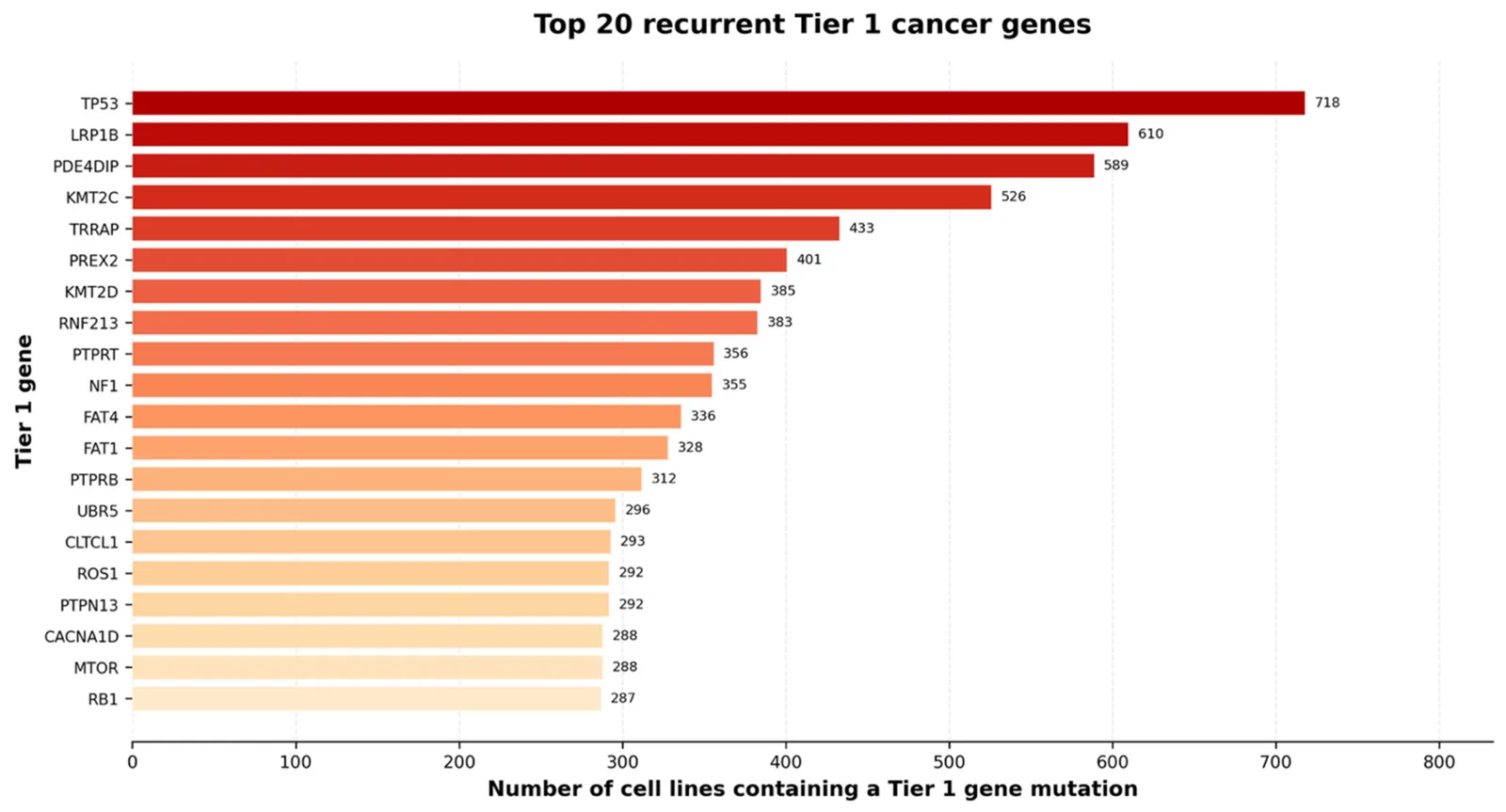
Top 20 recurrent Cancer Gene Census (CGC) Tier 1 genes across the COSMIC Cell Lines Project. Tier 1 genes are ranked according to the number of independent cancer cell lines harbouring at least one mutation in each gene. The numbers shown on the bars represent the total number of mutated cell lines for each Tier 1 gene. TP53 was the most recurrent Tier 1 gene, followed by LRP1B, PDE4DIP, KMT2C and TRRAP.

**Figure 4:**
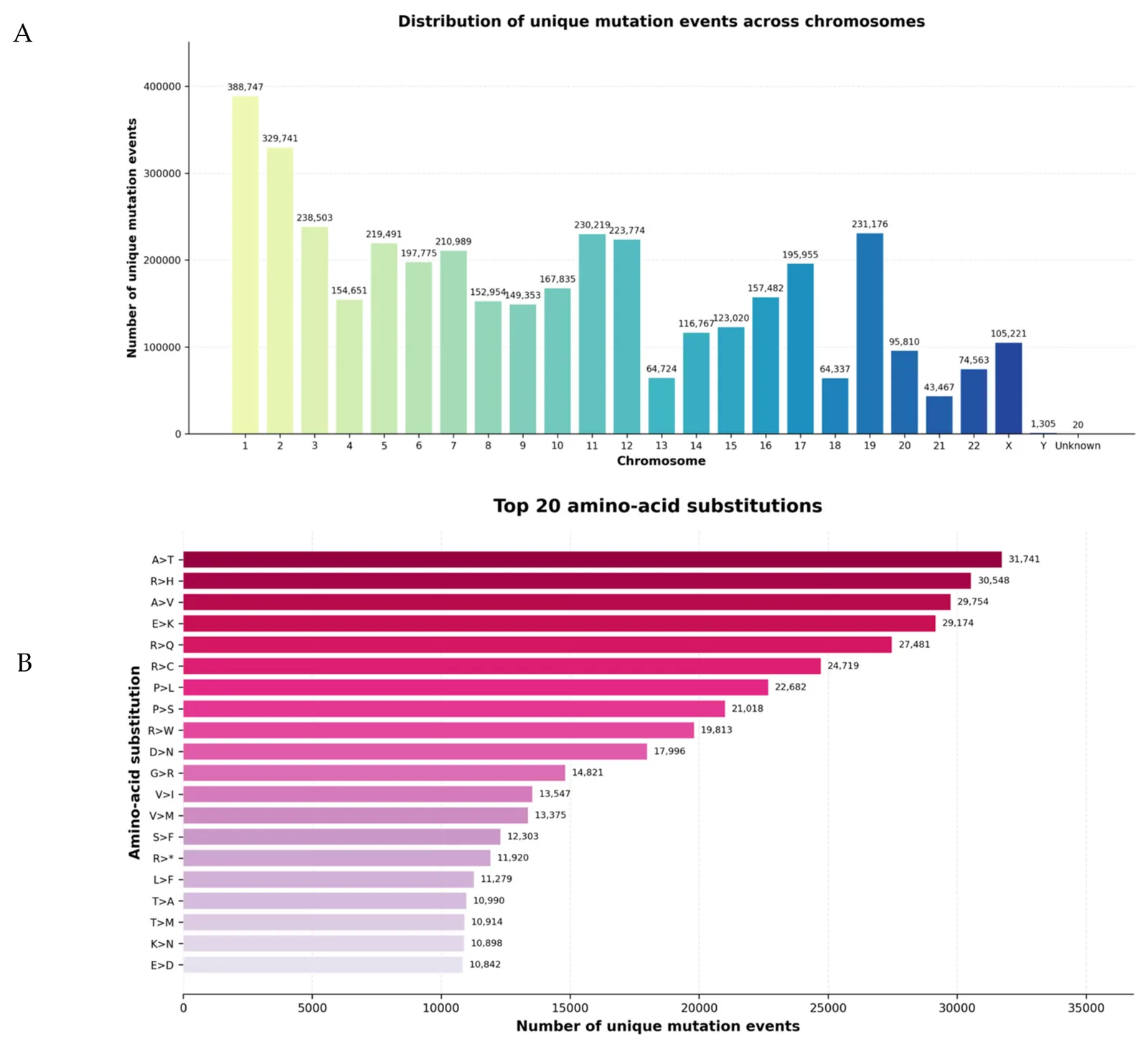
Genome-wide chromosomal distribution of somatic mutations and recurrent amino acid substitutions. (**A**) Distribution of unique mutation events across human chromosomes. (**B**) Top 20 amino acid substitutions ranked according to the number of unique mutation events observed across all analysed cancer cell lines. Values shown on the bars indicate the corresponding mutation counts.

**Figure 5:**
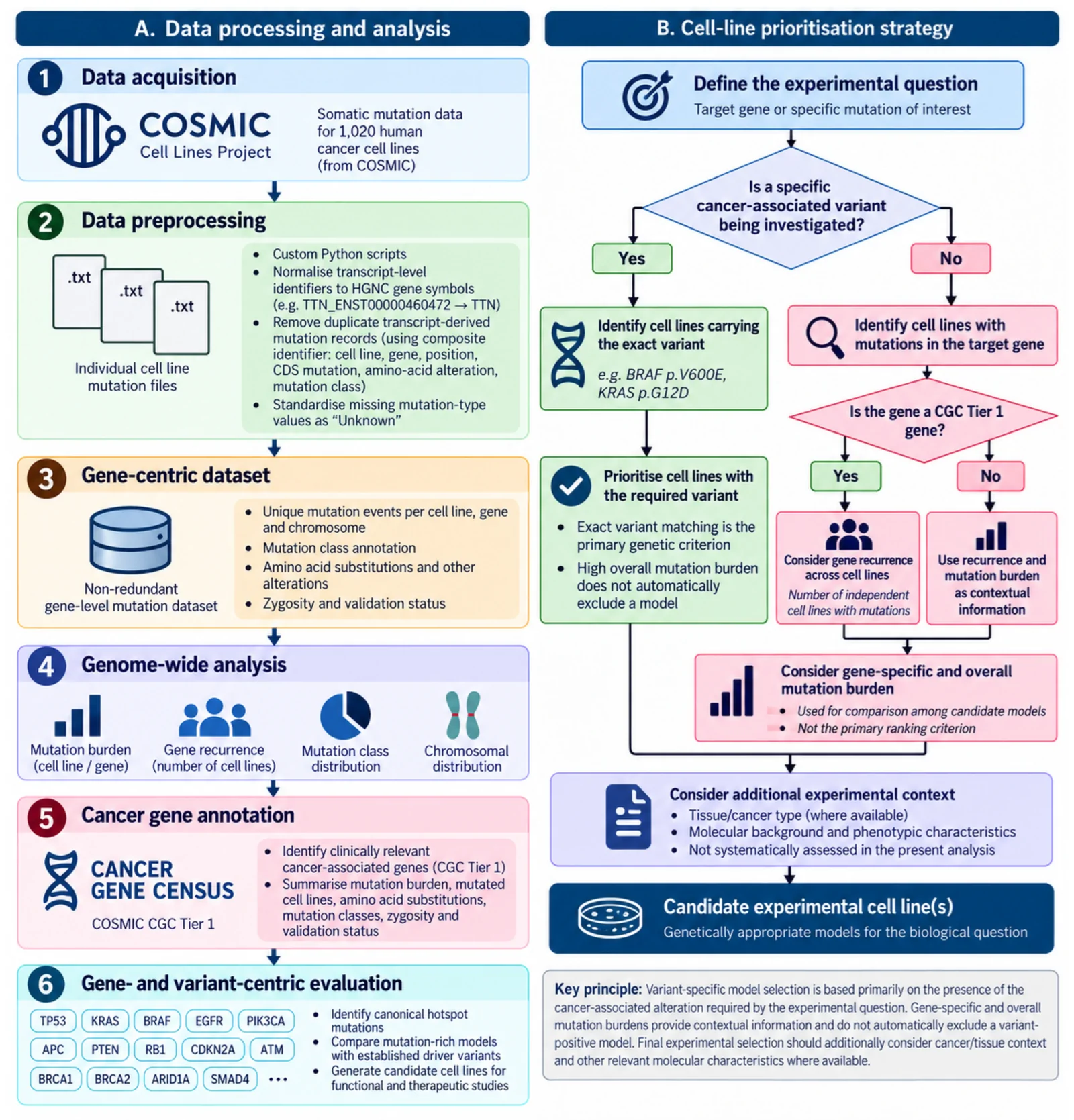
Gene- and variant-centric workflow for cancer cell-line analysis and experimental model prioritisation. Somatic mutation records from 1020 COSMIC cancer cell lines were normalised to gene symbols and deduplicated to generate a non-redundant gene-level dataset. Mutation burden, gene recurrence, mutation-class distribution, chromosomal distribution and Cancer Gene Census (CGC) Tier 1 genes were subsequently characterised. For experimental model selection, a hierarchical decision strategy was applied according to the biological question. When a specific cancer-associated driver variant was the experimental target, cell lines carrying the exact alteration were prioritised, with exact variant matching serving as the primary genetic criterion. Gene-specific and overall mutation burdens were considered contextual characteristics rather than primary ranking or exclusion criteria; therefore, a highly mutated cell line was not automatically excluded when it carried the required variant. For gene-level investigations without a predefined variant, cancer-gene relevance, recurrence and mutation burden provided additional contextual information. Final experimental model selection should additionally consider cancer/tissue context and other relevant molecular or phenotypic characteristics where available, which were not systematically assessed in the present analysis.

**Table 1:** Top 20 cancer cell lines by mutation burden after exclusion of Unknown mutation types.

Rank	Cell Line	All Mutation Events	Unknown Events	Unknown (%)	Events Excluding Unknown	Original Rank	Rank Change
1	SNU-1040	98,200	54,724	55.73	43,476	1	0
2	Daudi	67,635	26,754	39.56	40,881	2	0
3	CW-2	45,113	27,121	60.12	17,992	5	−2
4	SNU-81	56,687	40,254	71.01	16,433	4	0
5	KARPAS-45	38,528	22,636	58.75	15,892	7	−2
6	HCC2998	56,793	41,788	73.58	15,005	3	+3
7	HCT-15	40,264	25,276	62.78	14,988	6	+1
8	SNU-175	30,604	17,376	56.78	13,228	12	−4
9	Jurkat	28,021	15,288	54.56	12,733	14	−5
10	EN	31,449	18,998	60.41	12,451	11	−1
11	HT-115	38,327	25,909	67.60	12,418	8	+3
12	MOLT-4	26,707	14,550	54.48	12,157	17	−5
13	Mewo	36,343	24,217	66.63	12,126	9	+4
14	SW684	32,391	20,977	64.76	11,414	10	+4
15	GP5d	29,562	18,489	62.54	11,073	13	+2
16	MFE-319	27,702	17,447	62.98	10,255	15	+1
17	DND-41	21,190	11,049	52.14	10,141	22	−5
18	LS-411N	24,224	14,199	58.62	10,025	19	−1
19	OCI-AML5	17,142	7252	42.31	9890	27	−8
20	MCC13	27,142	17,520	64.55	9622	16	+4

**Table 2:** Cancer cell lines with the highest mutation counts in selected Cancer Gene Census Tier 1 genes.

Tier 1 Gene	Mutated Cell Lines (n)	Highest-Ranked Cell Line	Unique Mutation Events
*TP53*	718	GCT	4
*LRP1B*	610	SNU-81	42
*PDE4DIP*	589	SNU-1040	30
*KMT2C*	526	SNU-1040	26
*TRRAP*	433	SNU-1040	32
*PREX2*	401	Mewo	19
*KMT2D*	385	SNU-1040	26
*RNF213*	383	SNU-1040	15
*PTPRT*	356	SW684	17
*NF1*	355	Daudi	44

**Table 3:** Selected representative cancer-associated driver variants were used as examples to demonstrate variant-specific cell-line selection.

Pathway	Gene	Highest Mutation-Count Model	Canonical Mutation Found	Cell Line(s) Carrying the Canonical Mutation	Recommended Use
RAS/MAPK	*BRAF*	Farage, 8 variants	p.V600E	A375, ACN, BHT-101, G-MEL, HT-29, IGR-1, K2, LOXIMVI, LS-411N	Preferred models for *BRAF* V600E and RAF/MEK inhibitor studies
RAS/MAPK	*KRAS*	Daudi, 7 variants	p.G12D	GP5d, HEC-1, KP-4, A427, AGS, AsPC-1, CCRF-CEM	KRAS G12D functional studies
RAS/MAPK	*KRAS*	Daudi, 7 variants	p.G12V	CFPAC-1, CAPAN-1, NCI-H2291	KRAS G12V studies
RAS/MAPK	*KRAS*	Daudi, 7 variants	p.G12C	NCI-H2291	KRAS G12C-targeted studies
RAS/MAPK	*KRAS*	Daudi, 7 variants	p.G13D	Must be retrieved from all KRAS rows, not only the top-ranked rows	Colorectal KRAS G13D studies
RTK	*EGFR*	LS-411N, 9 variants	p.E746_A750del	HCC-827, PC-14	EGFR exon 19 deletion studies
RTK	*EGFR*	LS-411N, 9 variants	p.G719S	SW48	Uncommon activating EGFR mutation
PI3K/AKT	*PIK3CA*	HCC2998, 5 variants	p.E545K	HCT-15, TE-5, A388, HCC202	PIK3CA helical-domain studies
PI3K/AKT	*PIK3CA*	HCC2998, 5 variants	p.H1047R	BT-20, CAL-148	PIK3CA kinase-domain studies
PI3K/AKT	*PIK3CA*	HCC2998, 5 variants	p.H1047L	GP5d	Alternative kinase-domain model

## Data Availability

The original contributions presented in this study are included in the article/Supplementary Materials. Further inquiries can be directed to the corresponding author.

## Supplementary Materials

The following supporting information can be downloaded at: https://www.mdpi.com/article/10.3390/ijms27198636/s1.

## Abbreviations

The following abbreviations are used in this manuscript:

CCLE	Cancer Cell Line Encyclopedia
CDS	Coding sequence
CGC	Cancer Gene Census
CIViC	Clinical Interpretation of Variants in Cancer
COSMIC	Catalogue Of Somatic Mutations In Cancer
CRISPR	Clustered Regularly Interspaced Short Palindromic Repeats
DNA	Deoxyribonucleic acid
DSR	Deanship of Scientific Research
GDSC	Genomics of Drug Sensitivity in Cancer
HGNC	HUGO Gene Nomenclature Committee
ICGC	International Cancer Genome Consortium
MAPK	Mitogen-activated protein kinase
MEK	Mitogen-activated protein kinase kinase
MSI	Microsatellite instability
PI3K	Phosphoinositide 3-kinase
RAS	Rat sarcoma
RAF	Rapidly accelerated fibrosarcoma
RTK	Receptor tyrosine kinase
TCGA	The Cancer Genome Atlas
MUC16	Mucin-16
TTN	Titin
HYDIN	Axonemal central pair apparatus protein HYDIN
NEB	Nebulin
OBSCN	Obscurin
